# Ribosomal proteins: a novel class of oncogenic drivers

**DOI:** 10.18632/oncotarget.20802

**Published:** 2017-09-11

**Authors:** Sergey O. Sulima, Kim De Keersmaecker

**Affiliations:** Department of Oncology, KU Leuven, University of Leuven, Leuven Cancer Institute, Leuven, Belgium

**Keywords:** ribosome, ribosomopathies, RPL5, proteasome inhibitor, bortezomib

With a diameter of ~30 nm and a mass of ~4.3 MDa, human ribosomes are structurally and functionally sophisticated cytoplasmic giants. Half of this mass comes from the 81 ribosomal proteins (RPs) which, formerly erroneously believed to simply act as dispensable assistants of the catalytic ribosomal RNA (rRNA), are in many cases essential for ribosomal functioning. Ribosomes can mechanistically be viewed as nanomachines, with RPs and rRNA fulfilling the roles of bolts, gears, switches, and wires. These components must dynamically interact with one another to orchestrate a complex series of events necessary for proper gene expression, and it is now clear that improper function of this machine can have broad impacts on cancer progression.

The production of ribosomes can be compared to the manufacturing of cars - an intricate, hierarchical cellular assembly line provides a platform on which many different components are brought together to make a highly complex, fine-tuned and accurate machine. Congenital mutations in RPs disrupt this assembly process and result in a collection of disorders known as ribosomopathies. Several of these, including Diamond Blackfan anemia (DBA), dyskeratosis congenita, and Shwachman-Diamond syndrome, carry the risk to paradoxically progress from an initial phase of hypo-proliferative symptoms at early age (e.g. anemia, ataxia) to a phase of hyper-proliferative symptoms (cancer) at advanced age (reviewed in 1). This observation provided the first indications that RP defects might play a role in oncogenesis. More recently, we described somatic mutations in RPL5 (also known as uL18) and RPL10 (uL16) in T-cell acute lymphoblastic leukemia (T-ALL) [2], providing the first direct link between ribosomal protein lesions and cancer. This was just the tip of the iceberg, and alterations in additional RPs such as RPS15 (uS19), RPL11 (uL15) and RPL22 (eL22) have since been described in 10-40% of multiple tumor types (reviewed in 3). Furthermore, downregulation of RPL5, RPL11 and RPL22 was recently shown to accelerate tumor formation in transgenic mice and mouse tumor xenograft models [3, 4]. Many RP defects display specificity for either congenital or somatic diseases, or even a single particular disease entity. In contrast, RPL5 is a recurrent target for congenital mutations in the ribosomopathy DBA and somatic mutations in cancer, with 2% of T-ALL, 11% of glioblastoma, 28% of melanoma, 34% of breast cancer and ≥40% of multiple myeloma patients displaying deletions or mutations [2, 4, 5]. Fittingly adorning the head of the large subunit like a crown, RPL5 is thus the reigning king regarding ribosomal protein incidences implicated in cancer.

**Figure 1:**
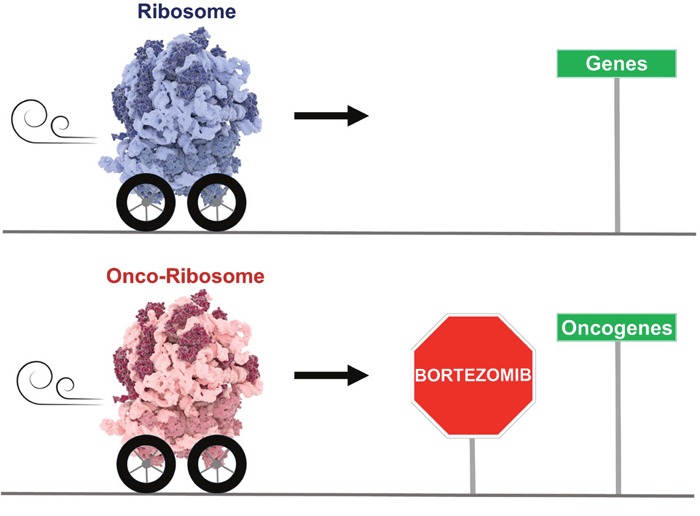
Ribosomal protein mutations in cancer rewire the ribosome and the cellular gene expression profile

The above studies quickly established mutations in ribosomal proteins as a novel, underexplored class of oncogenic factors. Early functional work suggests that, in addition to negatively impacting ribosome assembly similarly to RP mutations in ribosomopathies, the somatic RP mutations also influence ribosomal function. Many RPs, some mutated in cancer such as RPL10, contain mobile platforms which, akin to gears and switches, are used by the ribosome as transmitters of information in a complicated network of allosteric wiring [6]. Much like the gas pedal in an automobile, these proteins are linked to multiple functional centers of the machine. Mutations in RPs can modify the tuning and working scheme of the machine, causing a rewired ribosome to drive an altered gene expression program (Figure 1). It is tempting to speculate that the strategic location of RPL5 – part of a protein bridge to the small subunit and thus perfectly positioned to act as a transducer of information – might partially explain its high mutation and inactivation incidence in cancer: such defects are potentially more likely to alter coordination within the ribosome. Additionally, it is now emerging that ribosomes vary in composition between tissues and that different species of ribosomes can even be present within one cell [7]. This specialization of ribosomes may influence the vulnerability of a particular ribosome species to different RP defects and may contribute to the tissue specificity of cancers linked to mutations in particular RPs. Finally, several RPs (e.g. RPL5 and RPL11) also have extra-ribosomal roles in regulating prominent cancer genes such as TP53 and c-MYC. It is currently unclear to what extent the RP defects in cancer modulate these various ribosomal and extra-ribosomal functions. However, the outcome is an oncogenic rewiring of the cellular protein expression profile. Examples include the overexpression of the oncogenic JAK-STAT signaling cascade in the case of the RPL10-R98S mutation in T-ALL [8], elevated c-MYC expression upon RPL11 inactivation, and induction of stemness factor Lin28B by RPL22 inactivation [3].

It is becoming key to identify therapies for ribosome-defective cancer, and proteasome inhibitors are early promising candidates. These inhibitors are among the recent successful agents for multiple myeloma treatment, and there is now a need for novel biomarkers to identify patients with the highest potential benefit. In this context, we demonstrated that patients with a lower expression of RPL5 are more likely to respond to the proteasome inhibitor bortezomib [5]. This higher response rate leads to a survival benefit following treatment with bortezomib after relapse as well as directly after diagnosis. Importantly, T-ALL associated lesions in RPL10 have also been linked to increased sensitivity to proteasome inhibitors [8], suggesting that such inhibitors might benefit cancer patients with various RP defects. Uncovering the full role of the various RP defects in tumor progression and the underlying mechanisms is an exciting and important avenue for future research which will pave the way towards the development of new classes of clinical “onco-ribosome” inhibitors.
